# Stereopsis and visual acuity: Bilateral trifocal versus blended extended depth of focus and diffractive bifocal intraocular lenses

**DOI:** 10.3389/fmed.2022.1042101

**Published:** 2022-10-19

**Authors:** Meiyi Zhu, Wei Fan, Guangbin Zhang

**Affiliations:** ^1^Department of Ophthalmology, Eye Institute and Affiliated Xiamen Eye Center of Xiamen University, School of Medicine, Xiamen University, Xiamen, China; ^2^Fujian Provincial Key Laboratory of Corneal & Ocular Surface Diseases, Xiamen, Fujian, China

**Keywords:** stereopsis, visual acuity, trifocal intraocular lens, extended depth of focus intraocular lens, multifocal intraocular lens, cataract

## Abstract

**Purpose:**

To compare stereopsis and visual acuity (VA) between bilateral implantation of trifocal intraocular lenses (IOL) and blended implantation of an extended depth of focus (EDOF) IOL with a bifocal IOL.

**Methods:**

This is a non-randomized, prospective comparative study included 74 eyes of 37 patients who underwent phacoemulsification and bilateral implantation of AT LISA tri 839MP IOL (bilateral group; 21 patients) or blended implantation of Tecnis Symfony ZXR00 and Tecnis ZLB00 IOL (blended group; 16 patients). The primary outcomes were stereoacuity and binocular VA. The secondary outcomes were visual defocus curve, quality of life, and patient satisfaction. Follow-up was performed 3 months after the surgery.

**Results:**

The mean near stereoacuity was 49.76 ± 22.67 and 120.63 ± 90.94 seconds of arc (arcsec) in the bilateral and blended groups, respectively (*P* < 0.001). Near stereoacuity was positively correlated with VA difference of two eyes (r = 0.896, *P* < 0.001). The mean binocular uncorrected visual acuity at 40 cm, 80 cm, 5 m, and corrected distance visual acuity at 5 m of the bilateral and blended groups was not statistically significant different. The bilateral group had better VA at a vergence from −2.5 to −4.0 D. Both groups obtained high quality of life and patient satisfaction scores.

**Conclusion:**

The bilateral and blended groups achieved good binocular VA, quality of life, and high patient satisfaction. However, the near stereoacuity of the blended group was worse.

## Introduction

Since the widespread use of mobile devices, many people have shown an increased need for near and intermediate vision, and patients have hoped to obtain a full range of vision after cataract surgery. Multifocal intraocular lenses (IOL) can provide multiple foci, enabling patients to obtain high spectacle independence (1). There are several ways to achieve a good whole range of visual acuity (VA), such as bilateral implantation of trifocal IOL or blended implantation of different multifocal IOL (also called contralateral implant strategy) (2–4). The contralateral implant strategy aims to combine the advantages of different multifocal IOL to achieve good binocular visual performance. Previous research has shown that the Tecnis Symfony ZXR00, which is the most widely used extended depth of focus intraocular lenses (EDOF IOL), can provide good distance and intermediate vision but has some limitations in near vision performance (5, 6). The blended implantation of an EDOF IOL with a low-add power bifocal IOL is an effective method to realize good VA from far to near distance (7–9).

Stereopsis is an important part of binocular vision. It is the awareness of the relative distance of objects from the observer through binocular vision only and is based on retinal disparity (10). Although people possess good vision, they also need stereopsis to lead normal lives or work, especially people who perform operations, use microscopes, or conduct other fine activities (11, 12). For cataract patients, surgery is the best solution to their diseases and optical correction, as an IOL after cataract extraction can restore stereopsis (13). Many studies have confirmed that patients can restore normal stereopsis after multifocal IOL implantation, the pseudoaccommodation and multifocality-induced retinal blur do not worsen stereopsis (14, 15). Previous studies have shown that patients who used contralateral implant strategy could achieve good stereoacuity (9, 16), but one study has shown the worse stereoacuity after blended implantation of different add power bifocal IOL compared to bilateral implantation (17). In recent years, there has been growing concern about whether using the contralateral implant strategy would impair stereopsis. The current study aims to assess visual outcomes after bilateral implantation of a trifocal IOL (Carl Zeiss Meditec AT LISA tri 839MP) and blended implantation of an EDOF IOL (Tecnis Symfony ZXR00) with a bifocal IOL (Tecnis ZLB00), and compare the main clinical outcomes in stereoacuity and visual acuity.

## Materials and methods

### Study design

This was a non-randomized, prospective comparative study involving patients who underwent bilateral cataract surgery at the Xiamen Eye Center affiliated with Xiamen University, Xiamen, Fujian, China, from July 2021 to May 2022. Ethical clearance was obtained from the Ethics Committee of Xiamen Eye Center of Xiamen University, this study adhered to the tenets of the Declaration of Helsinki. The informed consent had been obtained from all patients participating in the study.

The type of lens to be implanted was determined by the patient individual choice. Patients were divided into two groups: bilateral group or blended group. The bilateral group consisted of patients who had bilateral implantation of trifocal IOL (Carl Zeiss Meditec AT LISA tri 839MP). The blended group consisted of patients who had implantation of an EDOF IOL (Tecnis Symfony ZXR00) in the dominant eye and a bifocal IOL (Tecnis ZLB00) in the non-dominant eye. We used the pinhole test to determine the dominant eye. Patients were excluded if they had any of the following: (1) angle kappa greater than 0.5 mm, (2) any ocular or systemic disease that could influence postoperative VA, (3) previous refractive surgery and/or any other ocular surgery history, and 4) intraoperative or postoperative complications.

### Lenses

The AT LISA tri 839MP (Carl Zeiss Meditec AG, Inc.) is single-piece, aspheric (−0.18 asphericity), diffractive trifocal lens. It has a 6.0 mm optic bench with a central trifocal zone over a diameter of 4.34 mm and a peripheral bifocal zone from 4.34 to 6.0 mm. The light distribution is 50, 20, and 30% for distance, intermediate, and near foci, respectively. The additions are + 3.33 D for near and + 1.66 D for intermediate at the IOL plane; in addition, it has a + 3.75 D add in its outer bifocal area.

The Tecnis Symfony ZXR00 (Johnson & Johnson Vision, Santa Ana, Inc.) is a single-piece, aspheric (−0.27 asphericity) EDOF IOL. The optical zone is 6.0 mm. It has a patented diffractive echelette design to form an elongated focal zone with an addition of + 1.75 D at the IOL plane. The posterior achromatic diffractive surface has an echelette design for correction of chromatic aberrations and contrast sensitivity enhancement, which forms a step structure whose modification of height, spacing, and profile of the echelette extends the depth of focus.

The Tecnis ZLB00 (Johnson & Johnson Vision, Santa Ana, Inc.) is a single-piece, aspheric (−0.27 asphericity), diffractive bifocal lens. The optical zone is 6.0 mm. The IOL incorporates a posterior diffractive multifocal optic pattern designed to provide both near and distance vision, with a near power of + 3.25 D.

### Surgical technique

Phacoemulsification was performed by a single experienced surgeon. The temporal clear corneal incision was 2.2 mm. Continuous curvilinear capsulorhexis was performed in surgery, and the size of the capsulorhexis was approximately 5.5 mm. Surgery was performed using a standard technique on an active-fluidic torsional phacoemulsification machine (Centurion Vision System, Alcon Laboratories, Inc.).

### Preoperative examination

A complete preoperative ophthalmological examination was performed, including biomicroscopy, fundoscopy, uncorrected distance visual acuity (UDVA) at 5 m, corrected distance visual acuity (CDVA) at 5 m, pupil diameter and corneal spherical aberration (Pentacam; Oculus, Inc.), angle kappa (iTrace; Tracey Technologies Corp., Inc.), axial length and corneal astigmatism (IOLMaster 700; Carl Zeiss Meditec AG, Inc.). The IOL power was calculated using the Barrett Universal II formula. All eyes were targeted for emmetropia.

### Postoperative examination

The postoperative examinations included uncorrected near visual acuity (UNVA) at 40 cm, uncorrected intermediate visual acuity (UIVA) at 80 cm, UDVA and CDVA at 5 m, manifest refraction. The defocus curve from + 1.0 D to −4.0 D in decrements of 0.5 D were evaluated under distance correction. The stereoacuity at near distance (40 cm), intermediate distance (80 cm), and far distance (5 m). Subjective outcomes included quality of life and patient satisfaction.

A Binoptometer 4P was used to assess the stereoacuity of the patients. The measuring method was designed based on the principle of polarized light, similar to that of Titmus. This stereotest has been proven to be a reliable method for measuring stereoacuity (18), and has been used to evaluate the stereoacuity of patients (19). A stereoacuity level of 60 seconds of arc (arcsec) or better is considered good stereoacuity (20), and 100 arcsec is the lowest limit of normal stereoacuity (13).

Quality of life was evaluated based on the Chinese version of the visual function index-14 (VF-12-CN), and some minor adjustments were made according to current living habits (21). The difficulty scale was graded as not difficult (100 score), slight (75 score), moderate (50 score), difficult (25 score), and inability to read due to vision problems (0 score). The questionnaire had 12 items, and the average score for each item was calculated separately (excluding the “not applicable” responses).

Patient satisfaction was assessed with a five-point Likert scale: very satisfied (100 score), satisfied (75 score), neither satisfied nor dissatisfied (50 score), dissatisfied (25 score), and very dissatisfied (0 score).

### Statistical analysis

Statistical analysis was performed using SPSS for Windows software (v. 26.0, IBM Corp). The normal distribution of variable was evaluated using the Shapiro-Wilk test. Normally distributed variables were compared between the two groups using an independent-sample *t* test. Non-normally distributed variables were compared between the two groups using the Mann-Whitney *U* test. Pearson’s correlation test was used to evaluate the correlation between the VA difference of two eyes and stereoacuity at near distance. A *P* value of less than 0.05 was considered statistically significant.

## Results

A total of 37 patients were enrolled. Follow-up was performed 3 months after the surgery. The bilateral group included 42 eyes of 21 patients, the mean age was 59.33 ± 5.89 years. The blended group included 32 eyes of 16 patients, the mean age was 61.69 ± 7.20 years. No statistically significant difference was found in age of the two groups (*P* = 0.281). The preoperative ocular characteristics are shown in Table 1.

**TABLE 1:** Descriptive measures for preoperative ocular characteristics of bilateral and blended groups.

Measurement	Bilateral group (AT LISA tri 839MP)	Blended group (ZXR00/ZLB00)	*P* value
UDVA (logMAR)			0.016
Mean ± SD	0.49 ± 0.41	0.67 ± 0.41	
Range	0.00 to 1.70	0.10 to 2.00	
CDVA (logMAR)			0.005
Mean ± SD	0.22 ± 0.33	0.41 ± 0.44	
Range	0.00 to 1.70	0.10 to 2.00	
Corneal astigmatism (D)			0.312
Mean ± SD	0.65 ± 0.39	0.56 ± 0.30	
Range	0.00 to 1.61	0.00 to 1.30	
Corneal spherical aberration (μm)			0.282
Mean ± SD	0.29 ± 0.12	0.32 ± 0.10	
Range	0.09 to 0.57	–0.03 to 0.55	
Axial length (mm)			0.027
Mean ± SD	23.50 ± 1.15	24.08 ± 1.02	
Range	21.30 to 26.04	22.33 to 26.24	
Pupil diameter (mm)			0.201
Mean ± SD	2.90 ± 0.38	2.73 ± 0.66	
Range	2.10 to 3.86	1.64 to 4.08	
Angle kappa (mm)			0.802
Mean ± SD	0.26 ± 0.13	0.23 ± 0.11	
Range	0.05 to 0.50	0.03 to 0.46	
IOL power (D)			0.158
Mean ± SD	21.25 ± 2.69	20.66 ± 2.35	
Range	14.50 to 25.00	15.00 to 24.50	
Target refraction (D)			0.078
Mean ± SD	–0.04 ± 0.10	–0.08 ± 0.10	
Range	–0.17 to 0.17	–0.24 to 0.16	

CDVA = corrected distance visual acuity; D = diopters; logMAR = logarithm of the minimum angle of resolution; SD = standard deviation; UDVA = uncorrected distance visual acuity.

### Stereoacuity

**FIGURE 1:**
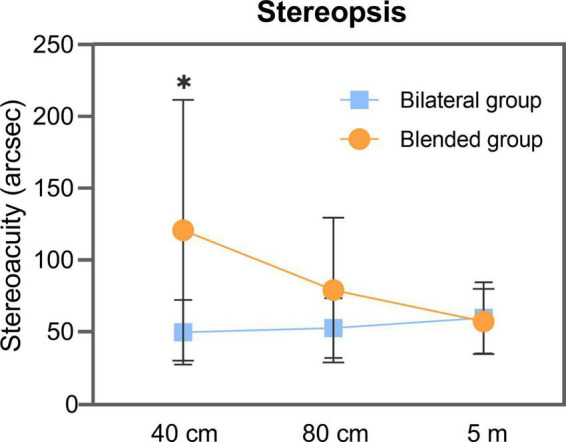
Stereoacuity measured by Binoptometer 4P of bilateral and blended groups at 40 cm, 80 cm, and 5 m distance. arcsec = seconds of arc. *Statistically significant difference between two groups.

For the bilateral group, the mean stereoacuity at near distance, intermediate distance, and far distance was 49.76 ± 22.67 (range 15 to 100), 52.62 ± 20.77 (range 30 to 100), and 59.76 ± 24.92 (range 30 to 100) arcsec, respectively. For the blended group, the mean stereoacuity at near distance, intermediate distance, and far distance was 120.63 ± 90.94 (range 45 to 400), 79.06 ± 50.41 (range 45 to 200), and 57.19 ± 22.66 (range 30 to 100) arcsec, respectively. No statistically significant difference was found between far and intermediate distance stereoacuity (*P* = 0.844, far distance; *P* = 0.083, intermediate distance), but a statistically significant difference was observed in near distance stereoacuity (*P* < 0.001) (Figure 1).

At far distance, good stereoacuity was achieved in 13 of 21 (62%) and 12 of 16 (75%) patients in the bilateral and blended groups, respectively. At intermediate distance, good stereoacuity was achieved in 17 of 21 (81%) and 11 of 16 (69%) patients in the bilateral and blended groups, respectively; all patients in the bilateral group had normal stereoacuity, whereas two patients in the blended group had abnormal stereoacuity (both 200 arcsec). At near distance, good stereoacuity was achieved in 17 of 21 (81%) and 4 of 16 (25%) patients in the bilateral and blended groups, respectively; all patients had normal stereoacuity in the bilateral group, whereas four patients had abnormal stereoacuity (three patients had 200 arcsec and one patient had 400 arcsec) in the blended group.

**FIGURE 2:**
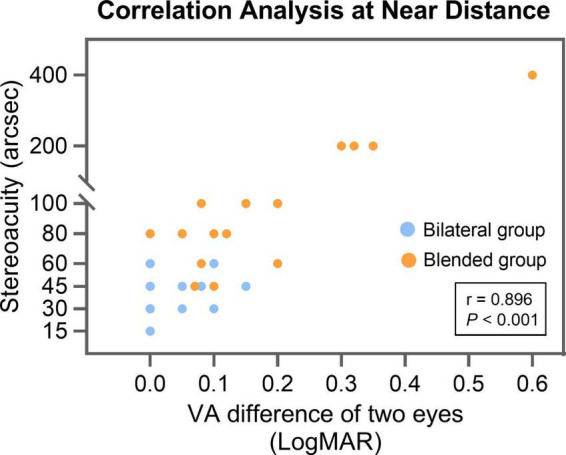
The correlation analysis between VA difference of two eyes and stereoacuity for all patients at near distance (40 cm). arcsec = seconds of arc; LogMAR = logarithm of the minimum angle of resolution; VA = visual acuity.

In near distance, the VA difference of two eyes of the bilateral and blended groups was 0.04 ± 0.06 and 0.18 ± 0.15 logMAR, respectively (*P* < 0.001). The correlation analysis indicated that the VA difference of two eyes was positively correlated with stereoacuity (correlation coefficient, *r* = 0.896, *P* < 0.001; Figure 2).

### Binocular visual acuity and manifest refraction

**FIGURE 3:**
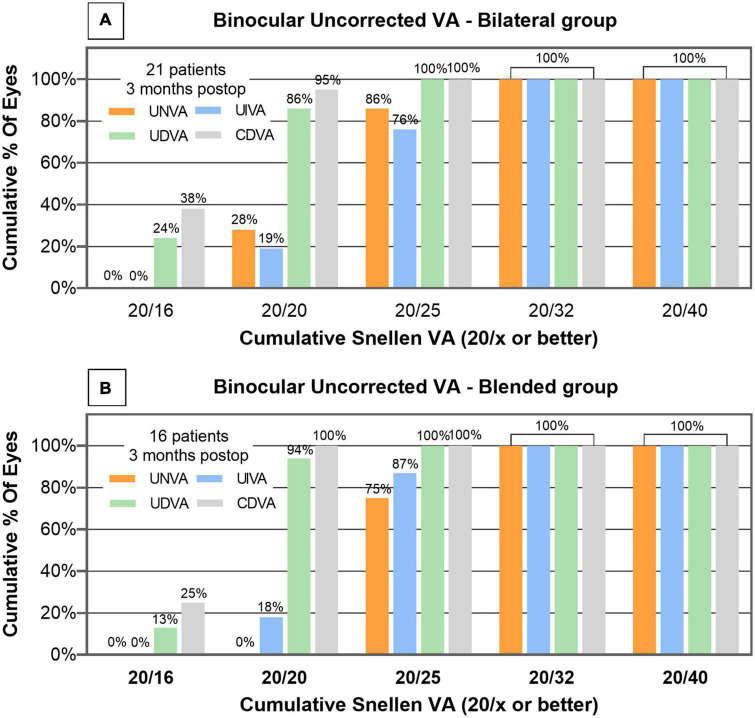
Distribution of postoperative binocular UNVA, UIVA, UDVA and CDVA of bilateral group **(A)** and blended group **(B)** measured 3 months after cataract surgery. corrected distance visual acuity = CDVA; uncorrected distance visual acuity = UDVA; uncorrected intermediate visual acuity = UIVA; uncorrected near visual acuity = UNVA; VA = visual acuity.

The mean binocular UNVA of the bilateral and blended groups was 0.08 ± 0.07 and 0.12 ± 0.05 logMAR (*P* = 0.101), respectively. The mean binocular UIVA of the bilateral and blended groups was 0.10 ± 0.07 and 0.09 ± 0.06 logMAR (*P* = 0.660), respectively. The mean binocular UDVA of the bilateral and blended groups was −0.01 ± 0.05 and 0.00 ± 0.04 logMAR (*P* = 0.868), respectively. The mean binocular CDVA of the bilateral and blended groups was −0.03 ± 0.05 and −0.02 ± 0.04 logMAR, respectively (*P* = 0.639). The proportion of patients in bilateral group with binocular UNVA, UIVA, UDVA, and CDVA of 0.1 logMAR (Snellen 20/25) or better was 86%, 76%, 100%, and 100%, respectively (Figure 3A). The proportion of patients in blended group with binocular UNVA, UIVA, UDVA, and CDVA of 0.1 logMAR (Snellen 20/25) or better was 75, 87, 100, and 100%, respectively (Figure 3B).

**FIGURE 4:**
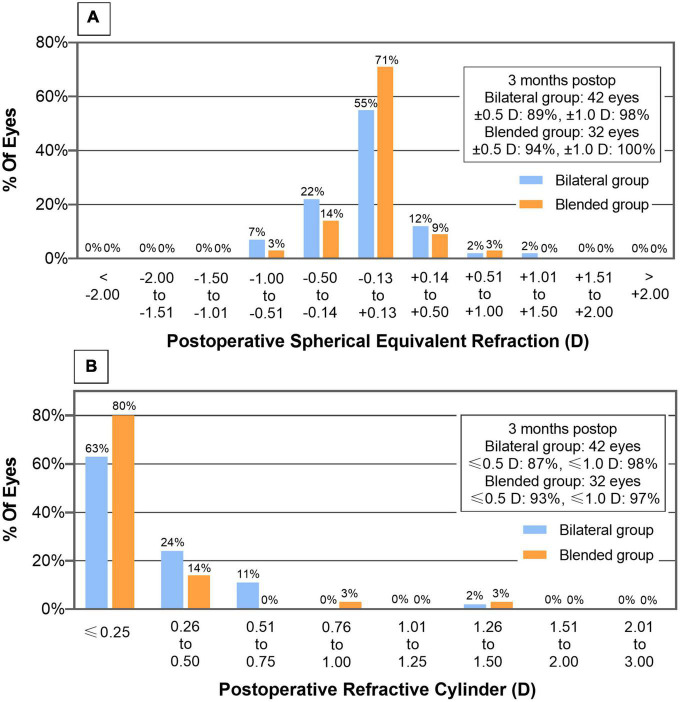
Distribution of postoperative spherical equivalent **(A)** and refractive cylinder **(B)** of bilateral and blended groups.

The mean spherical equivalent of the bilateral and blended groups was −0.05 ± 0.38 D and 0.00 ± 0.26 D, respectively (*P* = 0.450). The postoperative spherical equivalent was within ± 0.50 D in 89% of patients in the bilateral group and in 94% of patients in the blended group (Figure 4A). The mean postoperative cylinder of the bilateral and blended groups was −0.16 ± 0.40 D and −0.11 ± 0.35 D, respectively (*P* = 0.204; Figure 4B).

### Monocular and binocular defocus curves

**FIGURE 5:**
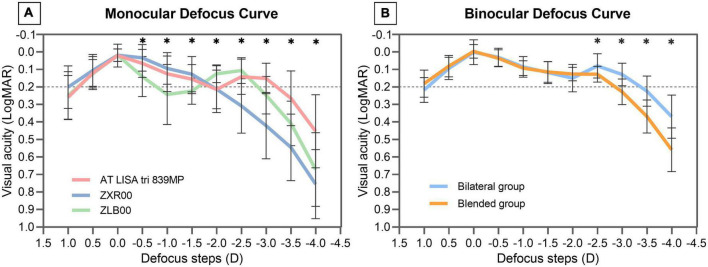
Monocular defocus curves of eyes implanted with AT LISA tri 839MP, ZXR00, and ZLB00 IOLs **(A)**. Binocular defocus curves of patients in bilateral and blended groups **(B)**. LogMAR = logarithm of the minimum angle of resolution. Results are shown in logMAR notation, with reference to the 0.2 logMAR thresholds; *Statistically significant difference between two groups.

Figure 5A illustrates the monocular defocus curves of eyes implanted with AT LISA tri 839MP, ZXR00, and ZLB00 IOLs. Among the three IOLs, no statistically significant difference was found at the defocus curves of + 1.0, + 0.5, and 0 D. At a defocus curve of −0.5, −1.0, and −1.5 D, AT LISA tri 839MP and ZXR00 were significantly better than ZLB00 (−0.5 D: *P* = 0.005 vs. AT LISA tri, < 0.001 vs. ZXR00; −1.0 D: *P* = 0.002 vs. AT LISA tri, < 0.001 vs. ZXR00; −1.5 D: *P* = 0.024 vs. AT LISA tri, 0.002 vs. ZXR00). No statistically significant difference was found between AT LISA tri 839MP and ZXR00. At a defocus curve of −2.0 D, ZLB00 was significantly better than AT LISA tri 839MP and ZXR00 (*P* = 0.006 vs. AT LISA tri, 0.048 vs. ZXR00). No statistically significant difference was observed between AT LISA tri 839MP and ZXR00. At a defocus curve of −2.5 D, AT LISA tri 839MP and ZLB00 were significantly better than ZXR00 (*P* < 0.001 both). No statistically significant difference was found between AT LISA tri 839MP and ZLB00. At a defocus curve of −3.0 D, AT LISA tri 839MP maintained good visual performance, but ZLB00 (*P* = 0.016) and ZXR00 (*P* < 0.001) were significantly poor. Additionally, ZLB00 had significantly better VA than ZXR00 (*P* = 0.030). At the defocus curve of −3.5 and −4.0 D, AT LISA tri 839MP remained significantly better than ZXR00 and ZLB00 (−3.5 D: *P* < 0.001 vs. ZXR00, 0.011 vs. ZLB00; −4.0 D: *P* < 0.001 vs. ZXR00, 0.005 vs. ZLB00). No statistically significant difference was found between ZXR00 and ZLB00.

Figure 5B illustrates the binocular defocus curves of the bilateral and blended groups. The defocus VA from + 1.0 to −2.0 D was not statistically significantly different between the groups. At the defocus of −2.5, −3.0, −3.5, and −4.0 D, the VA of the bilateral group was significantly better than that of the blended group (−2.5 D: *P* = 0.029; −3.0 D: *P* < 0.001; −3.5 D: *P* < 0.001; −4.0 D: *P* < 0.001).

### Quality of life and patient satisfaction

All patients filled out the questionnaire for this study. Table 2 shows the questionnaire used in this study. For the bilateral group, the mean near, intermediate, and far distance activities scores were 93.95 ± 10.18, 96.33 ± 7.06, and 99.11 ± 2.24, respectively. For the blended group, the mean near, intermediate, and far distance activities scores were 94.66 ± 8.30, 97.14 ± 6.54, and 100.00, respectively. No statistically significant difference was found between the two groups (*P* = 0.964, near distance activities; *P* = 0.820, intermediate distance activities; *P* = 0.476, far distance activities). The mean patient satisfaction score was 91.67 ± 14.43 for the bilateral group and 92.19 ± 11.97 for the blended group. Patient satisfaction score of the bilateral and blended groups was not statistically significantly different (*P* = 0.964).

**TABLE 2:** Questionnaire used in this study to evaluate the quality of life and patient satisfaction.

Question	Answer
**Near distance activities**	
Do you have difficulty reading small print, such as labels on medicine bottles?	1-5 scale^a^
Do you have difficulty reading newspaper or a book?	1-5 scale^a^
Do you have difficulty using mobile phone and identify the content?	1-5 scale^a^
Do you have difficulty filling out forms or signing names?	1-5 scale^a^
**Intermediate distance activities**	
Do you have difficulty using computer?	1-5 scale^a^
Do you have difficulty playing games such as mahjong, chess?	1-5 scale^a^
Do you have difficulty cooking?	1-5 scale^a^
Do you have difficulty doing fine handwork, such as sewing, crocheting?	1-5 scale^a^
**Far distance activities**	
Do you have difficulty watching television?	1-5 scale^a^
Do you have difficulty recognizing people when they are close to you?	1-5 scale^a^
Do you have difficulty going down stairs at night?	1-5 scale^a^
Do you have difficulty reading street signs?	1-5 scale^a^
Patient satisfaction	
How satisfied are you with your surgery outcomes?	1- 5 scale^b^

^a^ Difficulty of doing daily activities was rated on a scale of 1 to 5: 1 = not difficult; 2 = slight; 3 = moderate; 4 = difficult; 5 = inability to read due to vision problems.

^b^ Patient satisfaction was rated on a scale of 1 to 5: 1 = very satisfied; 2 = satisfied; 3 = neither satisfied nor dissatisfied; 4 = dissatisfied; 5 = very dissatisfied.

## Discussion

The contralateral implant strategy is used to achieve a full range of binocular VA, as bilateral implantation of a trifocal IOL (2, 8). However, this method has shortcomings. Eyes implanted with different multifocal IOLs would cause a VA difference between eyes at some visual distance, it could reduce the stereoacuity (13, 22). Hayashi et al. (17) reported that the stereoacuity of patients who had implantation of bifocal IOL with different near addition was worse than that of patients who had bilateral implantation of a trifocal IOL. As studies on whether the contralateral implant strategy could affect stereopsis are lacking, this topic should be studied further. In the present study, we set up two groups (the bilateral implantation of trifocal IOL group and the blended implantation of EDOF IOL with a bifocal IOL group) and compared their visual outcomes. Furthermore, we used an identical stereotest to evaluate near, intermediate, and far distance stereoacuity after cataract surgery, thus making the stereoacuity of different distances more comparable.

In our study, the bilateral and blended groups achieved good binocular VA in near, intermediate, and far distance. Aside from VA measured at fixed distance, the binocular defocus range (defined as VA greater than 0.2 logMAR) of the bilateral group reached nearly 3.5 D, and that of the blended group reached nearly 3.0 D. Both groups achieved satisfactory binocular VA from far to near distance. The bilateral group showed better VA at a vergence of −2.5, −3.0, −3.5, and −4.0 D. Previous study has reported a better VA at a vergence of −3.0 and −3.5 D of patients implanted with ZXR00 and ZMB00 IOL than trifocal IOL (8). It is worth noting that ZMB00 had an addition power of + 4.0 D at the IOL plane, this design enhanced near vision. In the present study, we used ZLB00 to compensate for near vision, and it still provided good near vision. For patients with a strong demand for near vision, a bifocal IOL with higher addition power is feasible.

In terms of stereopsis, most patients of the bilateral and blended groups achieved good far and intermediate distance stereoacuity. By contrast, the near stereoacuity of the bilateral group was still at a good level, but that of the blended group was significantly poor (only 25% patients achieved good stereoacuity). Patients implanted with trifocal IOL bilaterally showed excellent stereoacuity at various distances after the surgery, but implantation of an EDOF IOL with a bifocal IOL did not achieve similar outcomes.

As shown in previous study, the stereopsis is not affected by measuring distance, as it depends on the binocular disparity of the patient (23). However, in this study, the mean far and intermediate distance stereoacuity of blended group was normal, but the mean near distance stereoacuity was abnormal. It is worth noting that the VA difference between two eyes of the blended group was 0.18 ± 0.15 logMAR. When one eye received a blurred image, it would become difficult to fuse the images received by both eyes and affect the formation of a three-dimensional image (24, 25). The decrease in stereoacuity was greater when the VA difference between two eyes exceeded 0.1 logMAR (25). In the current study, we also found a positive correlation between the VA difference of two eyes and stereoacuity at near distance, and the results showed a strong positive correlation of the two variables. Aside from visual acuity, age also affects stereopsis, and it tends to deteriorate after 65 years (26). The mean age of the bilateral and blended groups is no more than 65 years, and no statistically significant difference was found between the two groups.

To assess the subjective experience of the patients, we used the Chinese version of the Visual Function Index-14 (VF-12-CN) questionnaire to evaluate quality of life, and this questionnaire is a reliable and valid tool to assess the visual function of Chinese patients (27). The bilateral and blended groups achieved high quality of life and the patients encountered no difficulty performing daily activities at various distances. Regarding patient satisfaction, the patients in the bilateral and blended groups achieved high satisfaction, and no patient in this study was dissatisfied with the postoperative visual performance.

Notably, the patients in the blended group had significantly worse near stereoacuity than the bilateral group, but no significant difference was found in the near distance activities and patient satisfaction scores between the two groups. The interpretation may be many of the near distance items in the VF-12-CN are directly dependent on VA, such as filling out forms, signing names, reading newspaper, and using mobile phone. In this study, both the bilateral and blended groups obtained good near binocular VA, and the uncorrected VA had a direct impact on visual quality and influence patient satisfaction (28). Additionally, stereopsis not only depend on binocular cues to perceive depth, but also can obtain from monocular depth cues (such as use of shadows, compare relative size, and relative defocus blur), and patients can compensate for loss of stereopsis by using these monocular depth cues (10).

This study has some limitations. One limitation is the absence of reading acuity and reading speed. Reading ability plays an important role in work and life. We did not evaluate reading ability in this study, so we are unable to conduct a comprehensive assessment of functional vision. Another limitation is we cannot examine the stereoacuity of patients with cataract preoperatively. Currently, there is no stereotest designed for cataract patients. Decreased contrast sensitivity due to cataracts and different degrees of cataract in both eyes may affect the accuracy of a clinically available stereotest. Therefore, we are unable to compare stereoacuity before and after the surgery.

In conclusion, the bilateral and blended groups achieved excellent binocular VA at all ranges of distance, all patients had high quality of life and patient satisfaction. Bilateral implantation of trifocal IOL restored good stereopsis at near, intermediate, and far distance after cataract surgery, but the near stereopsis of patients who underwent blended implantation of an EDOF IOL with a bifocal IOL was impaired. Further studies on the effect of contralateral implant strategy on stereopsis should be performed.

## Data availability statement

The raw data supporting the conclusions of this article will be made available by the authors, without undue reservation.

## Ethics statement

The studies involving human participants were reviewed and approved by Ethics Committee of Xiamen Eye Center of Xiamen University. The patients/participants provided their written informed consent to participate in this study.

## Author contributions

MZ: conceptualization, methodology, validation, formal analysis, investigation, data curation, writing-original draft, writing-review and editing, and visualization. WF: conceptualization, data curation, and investigation. GZ: conceptualization, methodology, resources, supervision, and project administration. All authors contributed to the article and approved the submitted version.
